# Clinical outcomes among unresectable, locally advanced, and metastatic cutaneous squamous cell carcinoma patients treated with systemic therapy

**DOI:** 10.1002/cam4.3146

**Published:** 2020-06-24

**Authors:** C. Lance Cowey, Nicholas J. Robert, Janet L. Espirito, Kalatu Davies, Jennifer Frytak, Israel Lowy, Matthew G. Fury

**Affiliations:** ^1^ Baylor University Medical Center Sammons Cancer Center Texas Oncology, P.A. Dallas TX USA; ^2^ Data, Evidence & Insights McKesson Life Sciences The Woodlands TX USA; ^3^ Regeneron Pharmaceuticals, Inc. Tarrytown NY USA

**Keywords:** chemotherapy, cutaneous squamous cell carcinoma, metastatic, retrospective study, skin cancer

## Abstract

Prior studies of conventional chemotherapy or epidermal growth factor receptor inhibitors for advanced (ie, locally advanced cutaneous squamous cell carcinoma [laCSCC] or metastatic [mCSCC]) cutaneous squamous cell cancer enrolled ≤ 40 patients. This retrospective, observational study assessed real‐world treatment patterns and clinical outcomes in patients with unresectable laCSCC or mCSCC using electronic health records of patients who initiated first‐line (1L) systemic treatment from 1 January 2008 to 31 December 2015, with follow‐up to 30 September 2017. The median duration of follow‐up from 1L treatment was 10.1 months (range 0.03‐67.6 months). Duration of therapy (DOT) and overall survival (OS) were assessed using Kaplan‐Meier analysis. Response rate was calculated as the proportion of patients who achieved physician‐assessed‐response. Eighty‐two patients were identified (17 laCSCC and 65 mCSCC). Median age at 1L treatment initiation was 75 years; 85% were male, 88% had an Eastern Cooperative Oncology Group performance status of 1, and 84% had received radiotherapy. The most common 1L regimens were carboplatin + paclitaxel (27%) and cetuximab monotherapy (24%). The median 1L DOT was 4.1 months for laCSCC and 2.3 months for mCSCC. The physician‐assessed response rate for 1L therapy was 17.6% for laCSCC, and 18.5% for mCSCC. The median OS from 1L treatment initiation was 16.2 months for laCSCC, and 15.3 months for mCSCC. Only 24 patients (29%) received second‐line therapy. This is the largest retrospective data set regarding patients with advanced CSCC treated with anticancer systemic therapy prior to approval of the anti‐programmed cell death‐1 antibody, cemiplimab. Efficacy was low in both laCSCC and mCSCC. These data provide historic benchmarks for outcomes in patients with advanced CSCC prior to Food and Drug Administration approval of cemiplimab‐rwlc.

## Graphical abstract

This retrospective study describes the limited efficacy of systemic therapy in a real‐world population of patients with locally advanced and metastatic cutaneous squamous cell carcinoma. The data provide historic benchmarks for outcomes of treatment for these patients prior to the approval of cemiplimab.

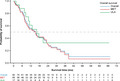

## 1 INTRODUCTION

Cutaneous squamous cell carcinoma (CSCC) is the second most common skin cancer, with nearly 400 000 cases in the United States in 2012.^1^ Risk factors include advanced age, male gender, history of chronic sun exposure, fair skin, and immunosuppression.^2^, ^3^ The vast majority of CSCCs of the skin are cured with local management such as wide local excision, Mohs surgery, or radiotherapy. However, a small percentage of patients develop advanced CSCC, a term that encompasses patients with metastatic CSCC (mCSCC) or with locally advanced CSCC (laCSCC) that is not amenable to curative surgery or curative radiation. There were approximately 3900‐8800 deaths due to advanced CSCC in 2012.^1^ In September 2018, cemiplimab‐rwlc, a monoclonal antibody directed against the programmed cell death (PD)‐1 receptor, became the first approved treatment for advanced CSCC by the United States Food and Drug Administration (FDA).^4^ The approval followed demonstration of safety and efficacy in a phase II study.^5^

Advanced CSCC can be responsive to cytotoxic chemotherapy and epidermal growth factor receptor (EGFR) inhibitors, although this is based on results of small single‐arm studies and case series. Two studies of platinum + 5‐fluorouracil–based chemotherapy enrolled 14 and seven patients with advanced CSCC, with reported response rates of 84% and 86%, respectively.^6^, ^7^ The triplet regimen of cisplatin + interferon alpha + 13‐*cis*‐retinoic acid (39 patients enrolled, 35 evaluable for response) showed a response rate of 34% among the evaluable patients, and the median overall survival (OS) was 14.6 months,^8^ and the regimen was not further developed. In an expert guideline document regarding CSCC, the panel members commented that the response rates reported in early chemotherapy studies in advanced CSCC were not replicated in subsequent studies.^9^

Epidermal growth factor receptor‐targeting agents have shown modest efficacy against advanced CSCC in single‐arm phase II studies that each enrolled 40 patients or fewer. In two studies of cetuximab and panitumumab, monoclonal antibodies directed against EGFR, the median OS times were 8.1 and 11 months, respectively.^10^, ^11^ Studies of gefitinib and erlotinib, EGFR tyrosine kinase domain inhibitors, reported median OS times of approximately 13 months among patients with advanced CSCC.^12^, ^13^

Retrospective studies of patients with advanced CSCC treated prior to 2012 also describe unmet need, but 25 patients^14^ and 32 patients^15^ received anticancer systemic therapies in these studies. To gain a larger experience in this patient population prior to the first approval of cemiplimab, the current study reviews the outcomes of patients with advanced CSCC who initiated treatment with commercially available anticancer systemic agents between 1 January 2008 and 31 December 2015 in The US Oncology Network.

## 2 PATIENTS AND METHODS

This was a retrospective, observational study of patients with laCSCC or mCSCC who received systemic therapy in The US Oncology Network. Patients had to have initiated systemic therapy between 1 January 2008 and 31 December 2015 and were followed through 30 September 2017. Patients were followed from initiation of first‐line (1L) systemic therapy until date of last patient contact, date of death, or the end of the study period (30 September 2017), whichever occurred first. Patients had variable lengths of follow‐up time available following their 1L therapy.

Data were collected by programmatic queries of McKesson Specialty Health's structured iKnowMed (iKM) electronic health record (EHR) database and manual chart review of unstructured iKM EHR data with abstraction onto case report forms. Vital status was supplemented with death dates from the Social Security Death Index.

The US Oncology Network is a physician‐led organization consisting of a network of integrated, community‐based oncology practices. iKM has been implemented across the network and captures outpatient practice encounter histories. The network is affiliated with approximately 1400 physicians in more than 60 community oncology practices across 25 states in the United States. This study was approved by the US Oncology institutional review board.

Included patients had to have a diagnosis of CSCC and have received systemic therapy for advanced CSCC that was not part of curative intent therapy; had to have an Eastern Cooperative Oncology Group (ECOG) performance status (PS) score of 0 or 1 at baseline; at least two visits within the US Oncology Network; and had to be ≥18 years of age at their first diagnosis of CSCC. Patients were excluded if they were enrolled in clinical trials at any time during the study period; had a concurrent other primary cancer diagnosis; had squamous cell carcinoma of unknown primary site or if the primary site of squamous cell carcinoma was the anogenital area; and if they received treatment with a PD‐1/PD‐ligand 1 inhibitor either prior to or during the study period.

Eligible patients were classified into the two study cohorts: unresectable laCSCC and mCSCC. Patients were defined as having laCSCC if they were not candidates for definitive radiation or surgery. Patients in the laCSCC cohort could not have nodal or distant metastatic disease except for nodal involvement due to direct invasion from the overlying skin. Patients with perineural involvement were included in the laCSCC cohort if involvement was due to direct extension that was noted on imaging (not discontiguous spread). If the imaging report was not available in the patient's chart, the patient was disqualified from the analysis. For inclusion into the mCSCC cohort, patients had to have evidence of metastatic disease based on TNM Classification of Malignant Tumors (TNM) staging, or a reported site of metastasis (including spread to regional lymph nodes, unless directly contiguous from overlying skin).

Descriptive statistics were used for demographics and clinical characteristics. Duration of therapy (DOT) and OS were analyzed by the Kaplan‐Meier method. Duration of therapy was defined as the time from initiation of therapy to treatment discontinuation for any reason, including censoring. OS was calculated from the start of systemic therapy. The physician‐assessed response to treatment was collected, and response rate was calculated as the proportion of patients who achieved a physician‐assessed‐response. The analyses were conducted using SAS^®^ (Version 9.4; SAS Institute Inc).

## 3 RESULTS

### 3.1 Patient characteristics

Among the 82 patients with advanced CSCC who met inclusion criteria (17 laCSCC and 65 mCSCC), the median age at the start of 1L treatment was 75 years, 85% were male, 79% were Caucasian, and 88% had an ECOG PS of 1 (Table 1). The median duration of follow‐up from 1L treatment was 10.1 months (range 0.03‐67.6 months). The most common primary site of disease was the head and neck, occurring in 67% of the patients. Prior treatments included surgery in 90% of the patients and radiotherapy in 84% of the patients. In the entire population, 8.5% of the patients were solid organ transplant recipients. Among the patients in the mCSCC cohort, the most common sites of metastatic disease were lymph nodes, lung, and bone.

**TABLE 1:** Baseline demographic and clinical characteristics among patients with cutaneous squamous cell carcinoma (CSCC), overall and stratified by metastatic and locally advanced disease

	Overall (N = 82)	mCSCC Population (n = 65)	laCSCC Population (n = 17)
Age at index (y)			
Median (Min, max)	75 (50, 90+)	76 (50, 90+)	69 (56, 89)
Age at index (y), n (%)			
≥50–<65	22 (26.8)	15 (23.1)	7 (41.2)
≥65–<75	19 (23.2)	17 (26.2)	2 (11.8)
≥75	41 (50.0)	33 (50.8)	8 (47.1)
Sex, n (%)			
Male	70 (85.4)	57 (87.7)	13 (76.5)
Female	12 (14.6)	8 (12.3)	4 (23.5)
Race, n (%)			
Black or African American	2 (2.4)	1 (1.5)	1 (5.9)
Caucasian	65 (79.3)	51 (78.5)	14 (82.4)
Not documented	8 (9.8)	8 (12.3)	0 (0.00)
Other	7 (8.5)	5 (7.7)	2 (11.8)
Practice location, n (%)			
South	60 (73.2)	47 (72.3)	13 (76.5)
West	17 (20.7)	13 (20.0)	4 (23.5)
Northeast	4 (4.9)	4 (6.2)	0 (0.00)
Midwest	1 (1.2)	1 (1.5)	0 (0.00)
ECOG performance status at index, n (%)			
0	8 (9.8)	8 (12.3)	0 (0.00)
1	72 (87.8)	55 (84.6)	17 (100.0)
Not reported	2 (2.4)	2 (3.1)	0 (0.00)
Primary CSCC site, n (%)			
Extremity—lower	4 (4.9)	4 (6.2)	0 (0.00)
Extremity—upper	14 (17.1)	13 (20.0)	1 (5.9)
Head/neck	55 (67.1)	40 (61.5)	15 (88.2)
Trunk	9 (11.0)	8 (12.3)	1 (5.9)
Sites of metastasis^a^, n (%)			
Regional lymph nodes (any)	49 (59.8)	49 (75.4)	0 (0.00)
Regional lymph nodes (only)	32 (39.0)	32 (49.2)	0 (0.00)
Lung	21 (25.6)	21 (32.3)	0 (0.00)
Liver	1 (1.2)	1 (1.5)	0 (0.00)
Bone^b^	16 (19.5)	15 (23.1)	1 (5.9)
Brain	1 (1.2)	1 (1.5)	0 (0.00)
Prior organ transplant, n (%)	7 (8.5)	6 (9.2)	1 (5.9)
Prior surgery reported for CSCC, n (%)	74 (90.2)	58 (89.2)	16 (94.1)
Standard excision, n (%)	67 (81.7)	53 (81.5)	14 (82.4)
Mohs micrographic surgery, n (%)	23 (28.0)	14 (21.5)	9 (52.9)
Cryosurgery, n (%)	1 (1.2)	1 (1.5)	0 (0.00)
Prior radiotherapy reported, n (%)	69 (84.1)	55 (84.6)	14 (82.4)

Abbreviations: ECOG, Eastern Cooperative Oncology Group; laCSCC, locally advanced CSCC; mCSCC, metastatic CSCC.

^a^ Patients may have had more than one site of metastasis.

^b^ One patient with laCSCC disease had a head/neck forehead lesion with invasion/direct extension to the bone.

### 3.2 Treatment patterns for 1L therapy

The most common 1L regimens among the overall population were carboplatin + paclitaxel (27%) and cetuximab (24%) (Table 2). These were also the most common 1L regimens among the mCSCC cohort (32.3% and 17%, respectively). Among the cohort with laCSCC, however, cetuximab was the most common 1L regimen, used by slightly more than half of the cohort (52.9%); followed by platinum + taxane combination regimens.

**TABLE 2:** First‐line (1L) treatments in the overall, metastatic, and locally advanced cutaneous squamous cell carcinoma (CSCC) populations

	Overall (N = 82)	mCSCC (n = 65)	laCSCC (n = 17)
1L treatments, n (%)			
Carboplatin + Paclitaxel	22 (26.8)	21 (32.3)	1 (5.9)
Cetuximab	20 (24.4)	11 (16.9)	9 (52.9)
Cisplatin + 5‐FU	6 (7.3)	6 (9.2)	0 (0.00)
Carboplatin + Cetuximab+Paclitaxel	5 (6.1)	3 (4.6)	2 (11.8)
Cisplatin	5 (6.1)	5 (7.7)	0 (0.00)
5‐FU + Cisplatin+Cetuximab	3 (3.7)	3 (4.6)	0 (0.00)
Other regimens^a^	21 (25.6)	16 (24.6)	5 (29.4)

Abbreviations: 5‐FU, 5‐fluorouracil; laCSCC, locally advanced CSCC; mCSCC, metastatic CSCC.

^a^ Other regimen combinations include those with n ≤ 2 overall: 5‐FU + carboplatin+cetuximab, capecitabine, carboplatin, carboplatin + 5‐FU, carboplatin + cetuximab, carboplatin + docetaxel+cetuximab, cetuximab + 5‐FU, cetuximab + capecitabine, cisplatin + capecitabine, cisplatin + paclitaxel, erlotinib, paclitaxel.

The median 1L DOT was 2.4 months (range, 0.03‐31.2) for the overall population, 4.1 months for the laCSCC cohort (range, 1.2‐22.1) and 2.3 months for the mCSCC cohort (range, 0.03‐31.2). The physician‐assessed response rates for 1L were 18.3% overall (15 responses/82 patients), 17.6% for the laCSCC cohort (3 responses/17 patients), and 18.5% for the mCSCC cohort (12 responses/65 patients). Among the 15 responding patients, the median DOT was 7.3 months (range, 4.2‐8.5 months). All patients had discontinued 1L therapy during the study period.

### 3.3 Overall survival from 1L treatment initiation

**FIGURE 1:**
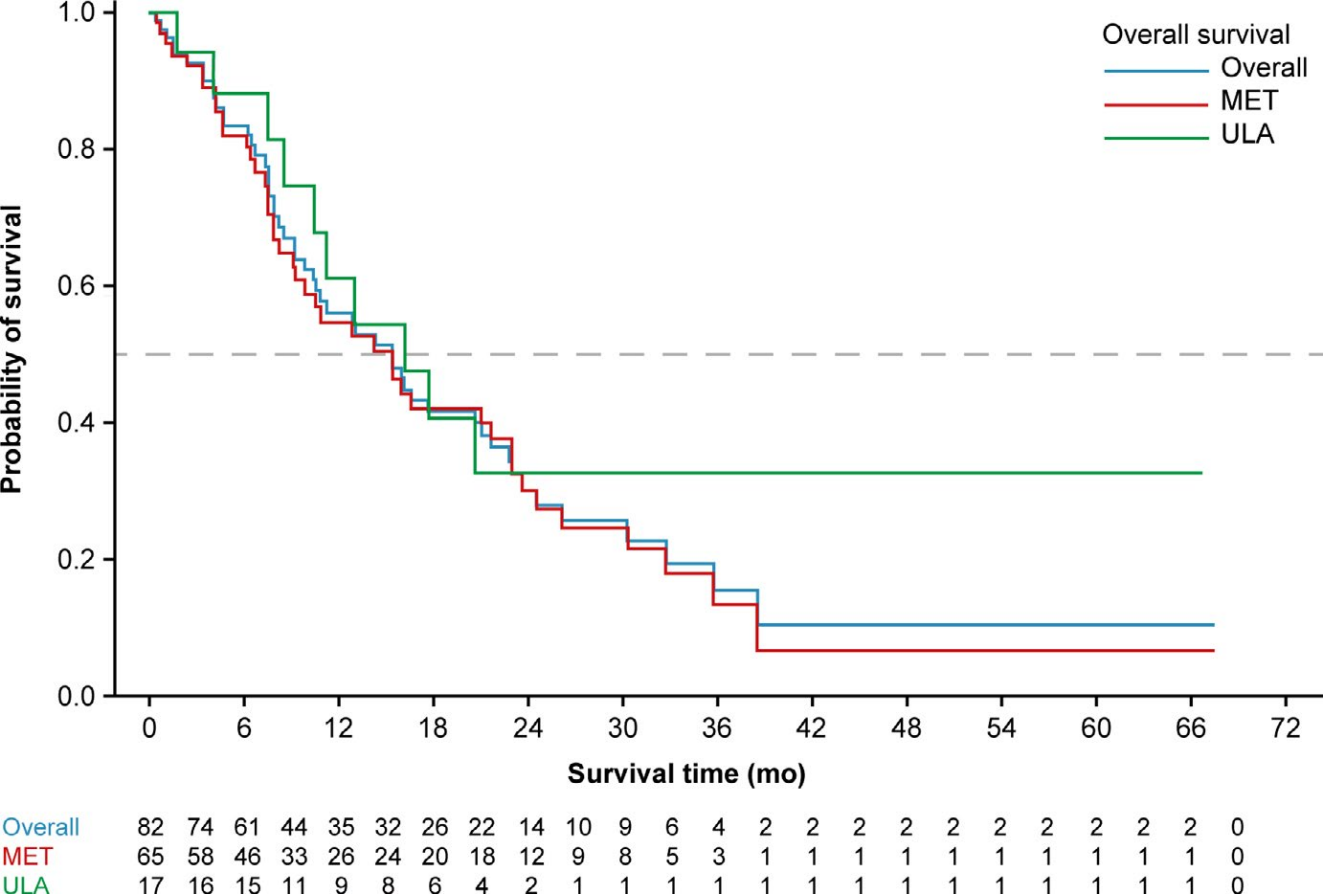
Kaplan‐Meier overall survival estimates from first‐line treatment initiation. MET, metastatic; ULA, unresectable locally advanced

Among 82 patients with advanced CSCC, there were 52 deaths during the study period (Table 3). The median OS from the start of 1L treatment was 15.3 months (95% CI, 10.4‐21.0) overall, 16.2 months (95% CI, 8.5 to not reached) for the laCSCC cohort, and 15.3 months (95% CI, 9.2‐22.9) for the mCSCC cohort (Figure 1; Table 3). The estimated OS at 12 months among all patients with advanced CSCC was 56.1% (95% CI: 43.6‐66.9) (Figure 1; Table 3).

**TABLE 3:** Kaplan‐Meier overall survival estimates from first‐line treatment initiation

	Overall (N = 82)	Study cohort	
		mCSCC (n = 65)	laCSCC (n = 17)
Events (%)	52 (63.4)	42 (64.6)	10 (58.8)
Median, mo (95% CI)	15.3 (10.4‐21.0)	15.3 (9.2‐22.9)	16.2 (8.5‐NR)
Survival probability, %			
6 mo	83.4 (73.2‐90.0)	82.1 (69.9‐89.7)	88.2 (60.6‐96.9)
12 mo	56.1 (43.6‐66.9)	54.8 (40.5‐66.9)	61.1 (32.8‐80.4)
18 mo	41.7 (29.7‐53.2)	42.1 (28.5‐55.1)	40.7 (16.9‐63.5)
24 mo	30.2 (19.1‐42.1)	30.2 (17.8‐43.5)	32.6 (11.1‐56.4)
36 mo	15.6 (6.3‐28.6)	13.5 (4.4‐27.6)	32.6 (11.1‐56.4)

Abbreviations: CI, confidence interval; laCSCC, locally advanced CSCC; mCSCC, metastatic CSCC.

### 3.4 Treatment patterns for second‐line therapy

Only 29% (20 out of 82 patients overall) received second‐line (2L) therapy: 31% in the mCSCC cohort and 24% in the laCSCC cohort. The most common 2L regimens in the overall population and in the laCSCC and mCSCC cohorts were cetuximab, carboplatin + paclitaxel, and docetaxel, respectively. The median 2L DOT was 3.4 months (range, 0.03‐27.7) for the overall population, 2.6 months for the laCSCC cohort (range, 0.03‐5.1), and 3.4 months for the mCSCC cohort (range, 0.95‐27.7).

## 4 DISCUSSION

This data set provides real‐world outcomes of 82 patients with advanced CSCC treated with commercially available anticancer therapy over a 7‐year period and is the largest experience available prior to the FDA approval of cemiplimab‐rwlc. The study population described here was comprised primarily of older Caucasian men with tumors arising in the head and neck, which is consistent with the known risk factors of advanced CSCC and provides reassurance that these results are representative of patients with this disease. For 1L therapy, the median DOT was 2.4 months, the physician‐assessed response rate was 18.3%, and the median OS was 15.3 months.

The patient demographics and outcomes from our study are similar to the retrospective analysis by the Dermatologic Cooperative Oncology Group (DeCOG) in Austria and Germany, in which 32 patients with advanced CSCC received anticancer systemic therapy.^15^ The baseline characteristics in the DeCOG study were similar to those reported here, although fewer patients had received prior radiotherapy in that study. Among these patients, a total of 39 lines of therapy were administered. The most commonly administered regimen was cetuximab, which comprised 38% (15/39) of the treatments. The remaining treatments in the DeCOG study comprised over a dozen different regimens, mostly platinum‐based. This result is consistent with the observation in the current study (Table 2) that there was no widely accepted standard of care for these patients, because a wide range of treatments were selected.

The 15.3‐month median OS in this study confirms the life‐threatening potential of both mCSCC and laCSCC. Table 4 summarizes the response rates and OS in previous studies of EGFR inhibitors or cytotoxic chemotherapy for patients with advanced CSCC. Several studies have reported higher response rates than those described in the current report. The variability of response rate assessments between studies may be attributed to multiple factors, including differing methods of response assessments and small sample sizes in previous studies. However, OS and DOT calculations are not subject to the same variability in methods as response assessments. As such, the 2.4‐month median DOT and the 15.3‐month median OS reported in this study are highly consistent with previously reported results regarding the limited efficacy of cytotoxic chemotherapy and EGFR inhibitors in the treatment of advanced CSCC.

**TABLE 4:** Efficacy outcomes in prior studies of advanced cutaneous squamous cell carcinoma with > 25 patients

Study	Regimen	n patients	Response rate (%)	OS, months
William et al^12^	Gefitinib	40	16	12.9
Maubec et al^10^	Cetuximab	36	28^a^	8.1
Gold et al^13^	Erlotinib	39	10	13
Shin et al^8^	Cisplatin + IFNα + retinoic acid	39	34^b^	14.6
Jarkowski et al^14^	Various^c^	25	44^c^	10.9
Hillen et al^15^	Various^c^	32^d^	26	NR^e^

Abbreviations: DeCOG, Dermatologic Cooperative Oncology Group; IFNα, interferon‐alpha.

^a^ Response rate in this study was per independent central review.

^b^ Among 35 patients evaluable for efficacy. Among all 39 enrolled patients the response rate was 31% (12 responders/39 patients).

^c^ Retrospective study.

^d^ Among 190 patients in the DeCOG study, only 32 received anticancer systemic therapy.

^e^ Overall survival (OS) among the patients who received anticancer systemic therapy was not reported (NR).

Clinical outcomes for advanced CSCC patients treated with cemiplimab‐rwlc were described after the study period for this report.^5^ Among 75 patients with metastatic CSCC, the objective response rate per independent central review was 46.7% (35 responders/75 patients). Among 10 patients with laCSCC, the objective response rate per independent central review was 60% (6/10 patients). Estimated median 12‐month OS was 81% among patients with metastatic CSCC in the pivotal phase II study of cemiplimab.^5^ Follow‐up of these patients continues regarding duration of response and OS. Long‐term follow‐up of these patients will be informative because, unlike conventional chemotherapy and EGFR inhibitors, cemiplimab's mechanism of action augments effector T‐cell function and has the potential for stimulating immune memory that can result in durable responses.

As a retrospective, observational EHR study, the limitations of the current report include missing and incomplete data. This can be due to services provided outside of the practice that were not reported or documented by the patient's physician, or due to differences in treatment patterns outside of clinical trials such as frequency of office visits and imaging tests. However, full review of patient charts was performed. Use of the EHR data represents usual care and can show real‐world findings, particularly in the setting where there is a lack of randomized trials.

## 5 CONCLUSION

This retrospective study confirms the limited efficacy of cytotoxic chemotherapy and EGFR inhibitors in a real‐world population that is twice as large as that of any prior study of these agents in the treatment of advanced CSCC. Efficacy was low in both the laCSCC and mCSCC cohorts. These data provide historic benchmarks for outcomes in patients with advanced CSCC prior to the approval of cemiplimab.

## CONFLICT OF INTEREST

CL Cowey reports honoraria from Regeneron Pharmaceuticals, Inc and institutional research funding from Regeneron Pharmaceuticals, Inc, Merck, Novartis, BMS, Genentech, Celldex, Array BioPharma, and Amgen. NJ Robert reports research funding from Side‐Out Foundation. He holds consulting or advisory roles with New Century Health, Bristol‐Myers‐Squibb, and Boehringer Ingelheim, and is an employee of McKesson Corporation. JL Espirito is an employee and shareholder of McKesson Corporation. K Davies is an employee and shareholder of McKesson Corporation. J Frytak reports travel expenses from Bristol‐Myers‐Squibb and is an employee and shareholder of McKesson Corporation. I Lowy is an employee and shareholder of Regeneron Pharmaceuticals, Inc. MG Fury is an employee and shareholder of Regeneron Pharmaceuticals, Inc.

## AUTHOR CONTRIBUTIONS

Conceptualization: CLC, NJR, JLE, JF, IL, and MGF; data analysis or interpretation and critical revision: CLC, NJR, JLE, KD, JF, IL, and MGF; drafting publication: JLE (http://annals.org/aim/article/2424869/goodpublication‐practice‐communicating‐company‐sponsored‐medical‐research‐gpp3).

## Data Availability

The raw data used for this analysis are not publicly available due to privacy or ethical restrictions.
